# Teaching and facilitating groups online: adapting to the COVID-19 pandemic

**DOI:** 10.1136/postgradmedj-2021-140619

**Published:** 2021-07-23

**Authors:** John Launer

**Affiliations:** Associate Editor, Postgraduate Medical Journal, London, UK

For many years I resisted doing any teaching online. A lot of my work involves facilitating discussions with people in small groups: commonly between 5 and 20 people. Nearly all of this work is interactive. Much depends on cultivating trust, intimacy and spontaneity. I assumed that the subtleties of interaction would be too hard to reproduce remotely. I had never heard of Zoom until March 2020 when the COVID-19 pandemic began to have an impact in the United Kingdom, nor had I used any similar platform. It was paradoxical to find that by the end of that month I was not only doing all my teaching remotely but was having to train other educators to do so too. Since then, I have become an unlikely but enthusiastic convert to teaching groups online. In this article I want to share some of what I have learnt, in the hope it might inform others who may not be finding this so enjoyable.

A pre-requisite for all good online teaching is technical slickness. When I first realised that the transition to working remotely was inevitable, I got colleagues to show me how they used different online platforms, and watched webinars from Harvard Business School.^1^ I also began to follow some of the emerging literature about successes in online teaching in my own field,^2–4^ as well as learning about experiences in other countries dealing with the pandemic.^5 6^ I then discovered that the quality of an event can improve even more if you do not try to do everything yourself, but enlist a separate ‘techie’ person who is slicker than you are. They can look after everything in the background while you do the teaching. I have sometimes asked IT personnel from the organisations I work for to do this, but I have also used participants attending an event who happen to have those skills too.

Not all platforms are equal. Whenever possible, I have learnt to insist on using ones that allow everyone in the group to be seen at the same time. (Some platforms like Microsoft Teams are not good for this unless you use breakout rooms all the time). Other technical tips include having safety nets for the worst contingencies, for example if you, a teaching colleague or a group member loses connection. Sometimes it can be useful to have two computer screens logged in so you can use them for different tasks. Having everyone’s mobile numbers or a WhatsApp group running in parallel with an online event can save the day.

## Ground rules and getting people to speak

It is worth having some ground rules about the functions you want people to use (like ‘chat’ or emojis), and also a quick tutorial at the beginning in how to use them – particularly the ‘mute’ button. Encouraging people to join from personal computers provides better quality than using phones. Some members of any group will always prefer not to be visible on screen. They may have legitimate reasons for this, or poor connectivity. All the same, I always urge participants to put their video on if at all possible because they will be so much more involved if everyone can see them. I ask them to have their correct names on display rather than a label like ‘iPad user’. As a facilitator I always use the ‘hide self view’ function if available, on the grounds that I would not normally be looking in a mirror while I teach. For reasons of confidentiality and safety, I would not advise allowing people to join a meeting from a car unless there are no other passengers and it is parked in the same place for the duration, with the engine off.

In face-to-face group teaching, I have always tried to intervene as little as possible so members have the maximum opportunity for interacting. Online, it can be more challenging to get people to speak. I have became bolder in calling out names and inviting individuals to contribute to the conversation. More recently, I have also been announcing at appropriate moments that I intend to withdraw from the discussion and let a group talk without any input from myself for the next ten or fifteen minutes. I explain that it is fine for the group to sit with periods of reflective silence if they want, just as they might in a live conversation. I then mute my audio and switch off my video. Although this can be unnerving for the group initially, it can also be liberating for everyone and help them to bond.^7^

Most groups seem to learn rapidly to self-monitor with regard to people interrupting or speaking over each other. Overuse of the chat function can be distracting, but it can also empower shyer people to join in the interaction while others are talking: these ‘dual’ exchanges can sometimes be an improvement on conventional group work. Another positive discovery has been how easy it is to put people into breakout rooms or to conduct a conversation in a ‘fishbowl’ format while others observe it. You can do the latter by telling the observers to switch off their videos for a while and go into ‘speaker view’ while the active participants stay visible. In some instances, this creates a greater sense of intimacy than doing working face-to-face. Two people talking in a virtual fishbowl, for example, will see only each other, without the distraction of a dozen or more faces staring at them as they would in a large room.

## Emotions and body language

While emotions and body language may not be as easy to read on a screen, I have been impressed by how quickly people can hone their skills to pick up cues such as intonation and hesitation. Many are also becoming more transparent in checking out ambiguous signals with questions like: ‘am I right in thinking you’re puzzled?' Even strong empathy can be expressed verbally by an expression like: ‘I wish I could give you a hug’. As experts in conversation analysis have pointed out, personal presence does not guarantee good communication, nor does distance prevent it.^8^

Everyone feels fatigue when working on a screen for hours on end, but sessions can usually be broken up into several shorter ones, or you can have more frequent breaks. Chats over coffee can be reproduced by suggesting that group members can invite two or three of their peers to do exactly that between formal sessions– even if it means brewing their coffee in four different kitchens. On occasions when I have witnessed the unscheduled appearance of toddlers and pets in teaching sessions, they have brought more joy than discomfiture to everybody present.

Remote interactions are not a like-for-like substitution for personal encounters and never will be. Yet my judgement after a year of working in this way is that there are ways of ameliorating or overcoming most of the problems I feared, or encountered at first. Before the pandemic, I would have predicted that my career as an educator would come to an end if I could only teach online. What has happened is the opposite. As well as carrying on with my usual work, I have delivered two virtual courses in California, done my first teaching in Saudi Arabia, and run a regular supervision group with attendees from Canada, Ireland, the UK and mainland Europe. People have joined these activities from the comfort of their own homes or offices, and I have done most of it while shielding in a house in rural Wales, interspersed with walks in the hills. Like everyone else, I am looking forward to hugging some of my colleagues for real once again, and to doing at least some of my teaching in person. Overall, I celebrate the gains that remote working has brought far more than I regret the losses.
